# Supplementary material for: First discovery of parasite eggs in a vertebrate coprolite of the Late Triassic in Thailand
Source: PLoS One. 2023 Aug 9;18(8):e0287891. doi: 10.1371/journal.pone.0287891 (PMC10411797; doi:10.1371/journal.pone.0287891)
Supplement: Attachment — Submitted filename: Response to reviewer.docx [file pone.0287891.s001.docx]

**Response to editor and reviewer**

**Additional Editor Comments:**

This review took some time, and I believe that the authors should be informed why it was so. I first sent the paper for review to two excellent specialists and received contrasting evaluations. One recommended rejection because the structure were not nematode eggs in their opinion, and the other recommended to accept the paper. Therefore I tried to obtain a third opinion. I also (confidentially) showed the photographs to a colleague who has practical experience with nematode eggs.

Finally I obtained three formal reviews, but it took time.

I suggest to follow the comments by Reviewer #3 about Ascarida vs Ascaroidea.

I also strongly suggest to add in the text a new paragraph explaining why the authors believe that these structures are not coccidian oocysts.

**Author’s Response:** We rewrite MS according referees suggestions including the possibility that some morphotypes are coccidian oocyst.

**Reviewers' comments:**

Reviewer's Responses to Questions

**Comments to the Author**

1. Is the manuscript technically sound, and do the data support the conclusions?

The manuscript must describe a technically sound piece of scientific research with data that supports the conclusions. Experiments must have been conducted rigorously, with appropriate controls, replication, and sample sizes. The conclusions must be drawn appropriately based on the data presented.

Reviewer #1: No

Reviewer #2: Yes

Reviewer #3: Yes

**Author’s Response:** We improved the interpretation of the photographs, So conclusions properly support our results.

2. Has the statistical analysis been performed appropriately and rigorously?

Reviewer #1: N/A

Reviewer #2: Yes

Reviewer #3: N/A

3. Have the authors made all data underlying the findings in their manuscript fully available?

The [PLOS Data policy](http://www.plosone.org/static/policies.action#sharing) requires authors to make all data underlying the findings described in their manuscript fully available without restriction, with rare exception (please refer to the Data Availability Statement in the manuscript PDF file). The data should be provided as part of the manuscript or its supporting information, or deposited to a public repository. For example, in addition to summary statistics, the data points behind means, medians and variance measures should be available. If there are restrictions on publicly sharing data—e.g. participant privacy or use of data from a third party—those must be specified.

Reviewer #1: Yes

Reviewer #2: Yes

Reviewer #3: Yes

4. Is the manuscript presented in an intelligible fashion and written in standard English?

PLOS ONE does not copyedit accepted manuscripts, so the language in submitted articles must be clear, correct, and unambiguous. Any typographical or grammatical errors should be corrected at revision, so please note any specific errors here.

Reviewer #1: Yes

Reviewer #2: Yes

Reviewer #3: Yes

5. Review Comments to the Author

Please use the space provided to explain your answers to the questions above. You may also include additional comments for the author, including concerns about dual publication, research ethics, or publication ethics. (Please upload your review as an attachment if it exceeds 20,000 characters)

**Reviewer** #1: Review of “First discovery of parasite eggs in a vertebrate coprolite of the Late Triassic in Thailand”

**Comment 1:** Unfortunately, there are no definite features that identify these structures as originating from nematodes. None have any indication of developing nematodes in them and the “shell” around the structure in fig.3 is not typical of the hardened, irregular surface of an ascaris egg. However, it is similar to an unsegmented sporoblast of a coccidian.

**Author’s Response:** We better categorized the different morphotypes observed in this study and provided further precisions and we included a systematic assessment in the discussion.

Note that unlike previous studies that discovered ascarids parasite egg in coprolithe, we are using thin section and we are not chemically extracting the parasites.In our method, the mamelonate structure of ascarids is not well observed as can be observed in chemical extraction technique, so images cannot be compared directly with studies that use chemical extraction instead thin section technique. We added this information in the new version of the manuscript. We presented new diagnostic features in our improved manuscript. As we rely on thin sections, the features we used to diagnose the parasites correspond to the egg features rather than to features of the embryos themselves. The ornamentation of eggs were mostly observed using different focus and can be checked on some morphotypes (ie. morphotype II and IV of our revised version where we brought more nuances, although we are confident with morphotype V, where the thickness of the shell falls in agreement with morphologies known in some Ascaroids.

**Comment 2:** The structures in figure 5 are also probably oocysts of coccidians, which are quite common today in the gut and feces of reptiles. One common genus is Eimeria, which has a number of species, some of which have oocysts that range from **42-82** µm in length and **27-54** µm in width, which overlap with the size of the figures in the paper.

**Author’s Response:** We agree with referee and we reorganized the different morphologies seen in the previous figures in a different way to better separate structures according to their size, ornamentation features, and shell thickness. We also added some sentence in our systematic assessment and suggested that these structures could be coccidian eggs according to size and thickness of the shell in the new version.

**Comment 3:** The structures in Fig.4 may not be of animal origin and have no resemblance to nematode eggs or coccidian oocysts.

**Author’s Response:** We reclassified the different organic structures observed in the coprolithe and modified completely the organization of our figures. Some previous structures could only be potentially be parasite eggs.Then our statement is much more nuanced. We also proposed that this morphotype (now morphotype IV) could be a plant spore or pollen although diagnostic features are also lacking.

**Reviewer #2:** The submitted manuscript presents the results of palaeoparasitological investigation of a vertebrate coprolite from Upper Triassic in Thailand. A total of three morphometrical types of round to oval formations, presumably helminth eggs, were recorded. Two of these types are considered to be characteristic of the eggs of ascaridoid nematodes, whereas the taxonomic status of the third-type eggs is unclear.

The manuscript is well written, the text is brief and clear, the results are well documented, so that I have no critical remarks to it. The results obtained are significant in that the findings represent the first record of helminth eggs in terrestrial vertebrate hosts from the Late Triassic in Asia. Therefore, I recommend this manuscript for publication.

**Reviewer** #3: This paper clearly presents identification of nematode eggs in a vertebrate coprolite.

**Comment:** The major comment relies on the choice of the chosen taxonomic level for the morphotype II. Because most traits are confusing (for example the thickness of the outer shell), how do the authors justify the choice to use the superfamily (Ascaridoidea) rather than the order (Ascaridida) to designate this morphotype?

**Author’s Response::** We reclassified the different structures differently to better make our systematic statement. We also agree with the comments made by Reviewer 3 that it is more appropriate to use Ascaridida, and therefore, we have made the necessary changes across all manuscript.

6. PLOS authors have the option to publish the peer review history of their article ([what does this mean?](https://mahasarakham-my.sharepoint.com/personal/59010261001_o365_msu_ac_th/Documents/ปีงบ%2065/งานวิจัย%2066/paper%20parasite%20edit%203/Resubmit/_blank)). If published, this will include your full peer review and any attached files.


If you choose “no”, your identity will remain anonymous but your review may still be made public.


**Do you want your identity to be public for this peer review?** For information about this choice, including consent withdrawal, please see our [Privacy Policy](https://www.plos.org/privacy-policy).

Reviewer #1: No

Reviewer #2: No

Reviewer #3: No

[NOTE: If reviewer comments were submitted as an attachment file, they will be attached to this email and accessible via the submission site. Please log into your account, locate the manuscript record, and check for the action link "View Attachments". If this link does not appear, there are no attachment files.]

While revising your submission, please upload your figure files to the Preflight Analysis and Conversion Engine (PACE) digital diagnostic tool, <https://pacev2.apexcovantage.com/>. PACE helps ensure that figures meet PLOS requirements. To use PACE, you must first register as a user. Registration is free. Then, login and navigate to the UPLOAD tab, where you will find detailed instructions on how to use the tool. If you encounter any issues or have any questions when using PACE, please email PLOS at [figures@plos.org](mailto:figures@plos.org). Please note that Supporting Information files do not need this step.
